# ﻿First description of the male leafhopper *Louanganastellata* Wei & Webb (Hemiptera, Cicadellidae, Deltocephalinae, Stegelytrini) from China

**DOI:** 10.3897/zookeys.1239.150264

**Published:** 2025-05-23

**Authors:** Keting Duan, Mick D. Webb, Jichun Xing

**Affiliations:** 1 Institute of Entomology, Guizhou University; The Provincial Special Key Laboratory for Development and Utilization of Insect Resources, Guizhou University, Guiyang, 550025, China Guizhou University Guiyang China; 2 Department of Life Sciences, Natural History Museum, Cromwell Road, London SW7 5BD, UK Natural History Museum London United Kingdom

**Keywords:** Auchenorrhyncha, identification key, morphology, new record, taxonomy

## Abstract

The monotypic leafhopper genus *Louangana* Wei & Webb, 2010 (Hemiptera, Deltocephalinae, Stegelytrini) is reported for the first time from China, and the male of its type species, *L.stellata* Wei & Webb, 2010, is described and illustrated for the first time. The characteristics of the genus *Louangana* are reviewed. The examined specimens are deposited in the Institute of Entomology, Guizhou University, Guiyang, China (GUGC).

## ﻿Introduction

**Figure 1.**
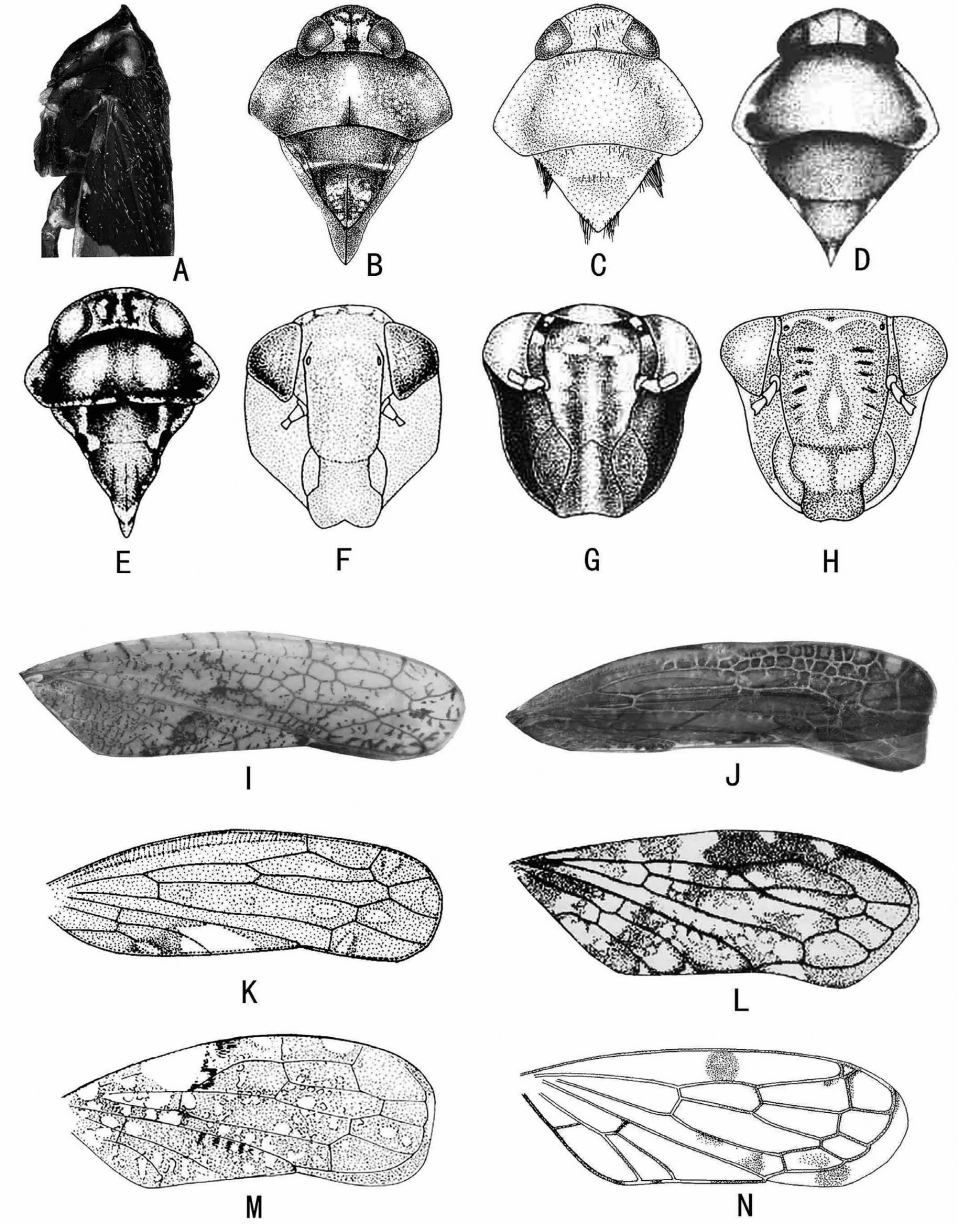
External features of Stegelytrini genera **A***Pachymetopiusfalcatus*: head and thorax, lateral view **B-E** head and thorax, dorsal view **B***Pseudododaorientalis***C***Cyrtaspinosa***D***Paracyrtadentata***E***Placidellusconjugatus***F-H** face, ventral view **F***Quiontugiafuscomaculata***G***Kunasianivosa***H***Trunchinusmedius***I-N** forewing **I***Sychentiahainanensis***J***Daochiareticulata***K***Wyuchivamenglaensis***L***Stenoloraabbreviata***M***Louanganastellata***N***Minucelladivaricata* (A after Wei et al. 2019: fig. 2; B after Zhang et al. 2007a: fig. 4A; C after Wei et al. 2008b: fig. 7B; D after Zhang et al. 2002b: fig.7A; E after Zhang et al. 2002a: fig. 1; F, M after Wei et al. 2010: fig. 16B, 12C; G after Zhang and Wei 2002: fig.2; H after Zhang et al. 2007b: fig. 58; I, J specimens in collection of Institute of Entomology, Guizhou University; K after Zhang et al. 2006a: fig. 5; L after Zhang et al. 2006b: fig. 3; N after Wei et al. 2008a: fig. 31).

Stegelytrini Baker, 1915 is one of the most morphologically diverse leafhopper groups found throughout Asia and the Pacific, and has two genera (*Stegelytra* Mulsant & Rey and *Wadkuptia* Linnavuori) found in the Mediterranean (Wei et al. 2010). The group may take its name from the ancient Greek “stegos” meaning “roof” for the slightly elevated roof-like outer margin of the forewing clavus (see lower arrow in Fig. 3B). Other distinguishing features, found in most genera, are the very spinose legs (Fig. 5A), very long antennae, antennae situated low on face (Fig. 1F–H), head relatively high in relation to the lateral pronotal carina in lateral view (Fig. 1A) and some unusual features found in some genera are a narrow head compared to the pronotum (Fig. 1B–E), lateral frontal sutures extending onto the top of the head (Fig. 1C, D); long scutellum compared to the pronotum (Fig. 1B, D, E) with one or more crests (see upper arrow in Fig. 3B), and the anteclypeus with a pair of stout apical setae. Also, in some genera the anteclypeus is greatly enlarged in the male and slightly enlarged in the female, and in these cases the clypellar suture is absent (Figs 3C, 5C). The enlarged clypellus in the male could be related to the feeding habit of some males in the group extracting chemicals from damp mud, referred to as mud-puddling (see Wei et al. 2010).

In Wei et al’s (2010) review of the tribe, three new monotypic genera were described only from females, something not normally contemplated, as leafhopper taxonomy is reliant mainly on characters of the male. However, this action was justified based on the distinctiveness of the taxa in question and relatively uncommon occurrence of Stegelytrini in collections, thereby making available some new characters for the group and improving knowledge of its morphological diversity. One such genus was *Louangana* Wei & Webb, 2010, described from a single female specimen of a new species, *L.stellata* Wei & Webb, 2010 from Laos. Recent studies on Chinese Stegelytrini revealed the male of this species, which is described and illustrated here for the first time.

## ﻿Material and methods

**Figure 2.**
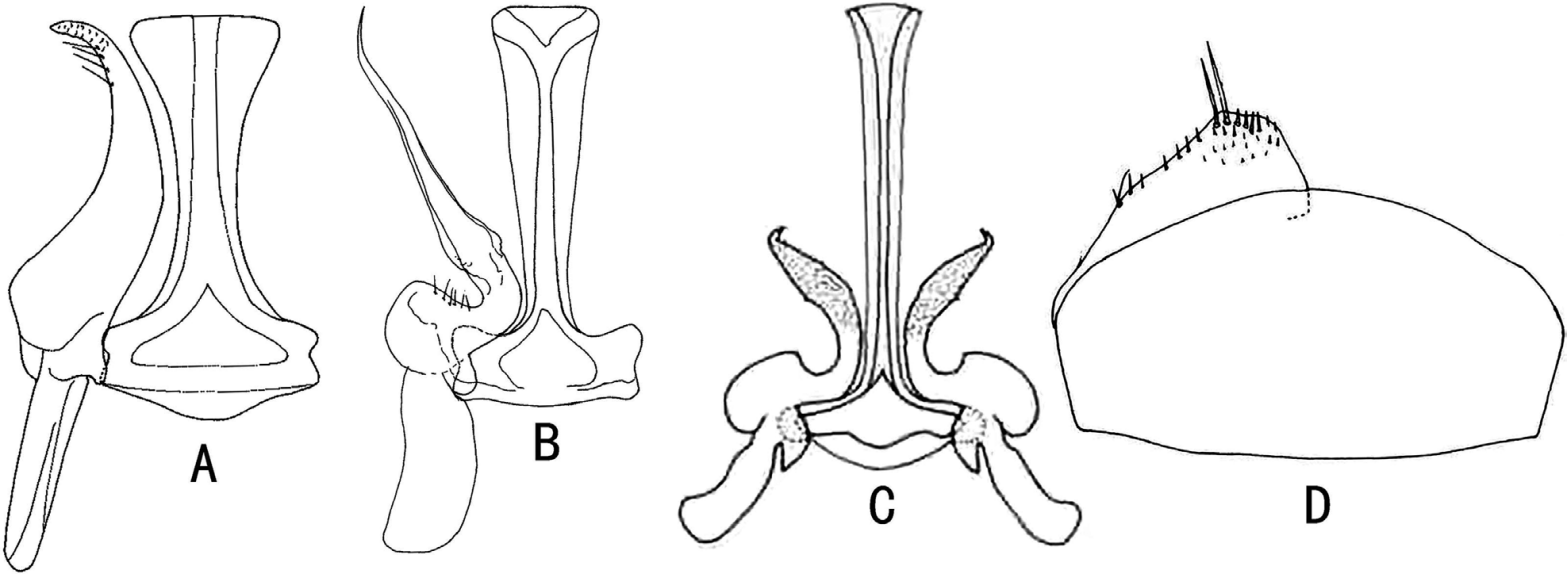
Male genitalia of Stegelytrini genera **A–C** style and connective, ventral view **A***Paradoxivenazhamuensis***B***Cyrtaspinosa***C***Paracyrtadentata***D***Trunchinusmedius*, valve and subgenital plate, ventral view (A after Wei et al. 2006: fig. 18; B after Wei et al. 2008b: fig. 7F; C after Zhang et al. 2002b: fig. 7E; D after Zhang et al. 2007b: fig. 63).

Specimens were used for the description and illustration. External morphology was observed under a stereoscopic microscope, and characters were measured with an ocular micrometer. The genital segments of the examined specimen were macerated in 10% NaOH. Color pictures for the adult habitus and the genitalia of specimens were obtained by KEYENCE VHX-6000 system and imported into Adobe Photoshop CS8 for labeling and plate composition. Reproduced line figures are used from various sources (see captions for Figs 1, 2 and Acknowledgements).

**Figure 3.**
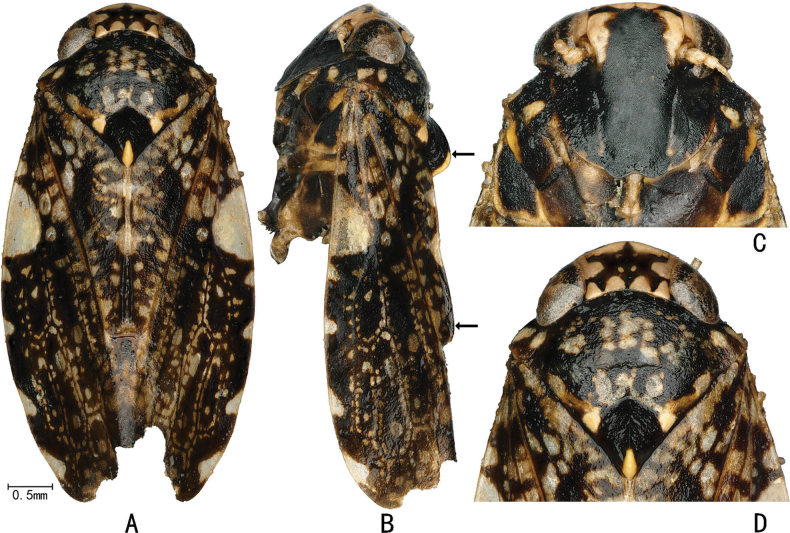
External features of *Louanganastellata* (female) **A** habitus, dorsal view **B** habitus, lateral view (upper arrow indicates scutellum crest, lower arrow indicates elevated claval margin **C** face, ventral view **D** head and thorax, dorsal view.

The morphological terminology used in the descriptions mainly follows Wei et al. (2010) and Li et al. (2011). Absolute measurements, in millimeters (mm), are reported for the body. All examined specimens are deposited in the Institute of Entomology, Guizhou University, Guiyang, China (GUGC).

### ﻿Key to Chinese genera of Stegelytrini (modified from Wei et al. 2010)

**Table:** 

1	Forewing veins reticulate (Fig. 1I, J)	**2**
–	Forewing veins not reticulate	**3**
2	Forewing apex rounded with narrow appendix (Fig. 1I)	***Sychentia* Wei & Webb, 2010**
–	Forewing apex truncate with broad appendix (Fig. 1J)	***Daochia* Wei, Zhang & Webb, 2006**
3	Forewing appendix indistinct (Fig. 1K)	***Wyuchiva* Zhang, Wei & Webb, 2006**
–	Forewing appendix distinct	**4**
4	Crown at intersection with face forming a ridge (Fig. 1A)	***Pachymetopius* Matsumura, 1914**
–	Crown rounded at intersection with face not forming a ridge	**5**
5	Scutellum with longitudinal medial ridge or keel or with ridge on each side posteriorly	6
–	Scutellum without longitudinal ridges or keel	**11**
6	Pronotum with medial longitudinal ridge basally (Fig. 1B)	***Pseudododa* Zhang, Wei & Webb, 2007**
–	Pronotum without medial longitudinal ridge	**7**
7	Scutellum with ridge on each side posteriorly (Fig. 1E)	***Placidellus* Evans, 1971**
–	Scutellum with long medial ridge posteriorly	**8**
8	Anteclypeus with lobe on each side apically (Fig. 1F)	***Quiontugia* Wei & Zhang, 2010**
–	Anteclypeus parallel margined or slightly widened apically, not forming a lobe on each side	**9**
9	Anteclypeus narrow basally and strongly broadening apically (Fig. 1G)	***Kunasia* Distant, 1908**
–	Anteclypeus broad basally with lateral margin slightly sinuated from base to apex	**10**
10	Forewing with apical margin oblique; basal cell elongate (Fig. 1L)	***Stenolora* Zhang, Wei & Webb, 2006**
–	Forewing with apical margin rounded; basal cell short and broad (Figs 1M, 3B, 5B)	***Louangana* Wei & Webb, 2010**
11	Forewing with very small outer apical cell (Fig. 1N)	***Minucella* Wei, Zhang & Webb, 2008**
–	Forewing without very small outer apical cell	**12**
12	Forewing veins bicoloured brown intervened with yellowish white; style apical process long (Fig. 2A)	***Paradoxivena* Wei, Zhang & Webb, 2006**
–	Forewing veins unicolourous; style apical process short to long	**13**
13	Anteclypeus broad basally (Fig. 1H); subgenital plate very short (Fig. 2D)	***Trunchinus* Zhang, Webb & Wei, 2007**
–	Anteclypeus narrow basally and expanded to apex; subgenital plate long	**14**
14	Scutellum with tufts of setae on lateral margin (Fig. 1C); style longer than connective (Fig. 2B)	***Cyrta* Melichar, 1902**
–	Scutellum without tufts of setae on lateral margin (Fig. 1D); style shorter than connective (Fig. 2C)	***Paracyrta* Wei, Webb & Zhang, 2008**

## ﻿Taxonomy

### ﻿ Louangana

Taxon classificationAnimaliaHemipteraCicadellidae

Wei & Webb, 2010

A6DD5B42-8AAD-591C-99E0-F6C25219CF18

Louangana Wei & Webb (in Wei et al.), 2010: 32.

#### Type species.

*Louanganastellata* Wei & Webb, 2010.

#### Description.

Body dark chocolate-brown, with small yellowish-white spots. Head small, distinctly narrower than pronotum; ocelli on anterior margin; antennae arising near lower corner of eye in facial view; laterofrontal sutures extending laterad of and beyond ocelli; vertex with fore margin slightly curved with fore and hind margins subparallel, medial length shorter than width between eyes; anteclypeus broader in male with gena narrower, with a pair of stout apical setae; clypellar suture absent. Pronotum with faint transverse striations. Scutellum well developed, slightly longer than pronotum, with medial longitudinal crest posteriorly, higher than pronotum. Forewing with outer margin of clavus elevated. Legs densely setose. Fore femur row AM with one stout seta and several irregularly arranged additional setae basally; AD and PD setal rows with short setae in basal half and long setae distally, few additional setae between longer setae, and row AV with several short setae. Fore tibia with dorsal setal formula obscured by the presence of several scattered macrosetae with more distal setae longer; 16 macrosetae in row AV and 15 macrosetae in row PV with numerous macrosetae decreasing in length toward base. Hind femur broadened distally and slightly bowed; apical setal formula 2+2+2+1+1+1. Hind tibia flattened and bowed, with all macrosetae approximately equal in length; many short and dense setae between AD and PD rows.

**Male and female genitalia.** See species description.

#### Distribution.

Oriental region (Laos, China).

#### Remarks.

The discovery of the male of *Louanganastellata* reveals that the male anteclypeus is broader in the male than the female, with gena narrower (compare Figs 3C with 5C), a feature found in some other Stegelytrini (see Introduction). In addition, several distinctive features of the male genitalia are present, i.e., the subgenital plate is without macrosetae (Fig. 6B), the aedeagus is narrow throughout length in lateral view, without a basal apodeme and the basal half of the aedeagus is expanded laterally and lamellate (Fig. 6C–E) and the connective has its arms upturned (Fig. 6D).

The distribution of *Louangana* in Laos and Guizhou Prov. (China) suggests the genus may also be present in Yunnan Province (China), located between the two.

### ﻿ Louangana stellata

Taxon classificationAnimaliaHemipteraCicadellidae

Wei & Webb, 2010

A2E8ED49-39AD-5090-BFD3-AB8351D9D39B

Figs 3 , 4 , 5 , 6

Louangana stellata Wei & Webb (in Wei et al.), 2010: 33.

#### Description.

Head yellow with a five-pointed star-like mark centrally on vertex, more conspicuous in female (Figs 3D, 5D). Forewing with a large white transparent patch against costal margin sub-basally (Fig. 3A, B).

#### External features as in generic description.

**Male genitalia.** Pygofer side wide basally, apex narrow, with numerous macrosetae on posterior area (Fig. 6A). Anal tube short (Fig. 6A). Valve approximate trapezoid, nearly 2.5× as broad as median length (Fig. 6B). Subgenital plate subtriangular, without macrosetae along lateral margin, length of outer margin about 2.2× width of base (Fig. 6B). Aedeagus sinuate in lateral view, without basal apodeme, expanded laterally and lamellate in basal half, shaft tubular, gonopore apical (Fig. 6C–E). Connective articulated with aedeagus, ‘T’ shaped in ventral view with stem nearly two-thirds length of arms, arms upturned (Fig. 6C–E). Style elongate, outer basal arm long and tapered to apex, inner basal arm very short; apical process strongly curved basally and tapered to acute apex; preapical lobe relatively long, lying parallel to apical process (Fig. 6F). Dorsal connective present in phragma adjacent to base of aedeagal shaft (Fig. 6D).

**Figure 4.**
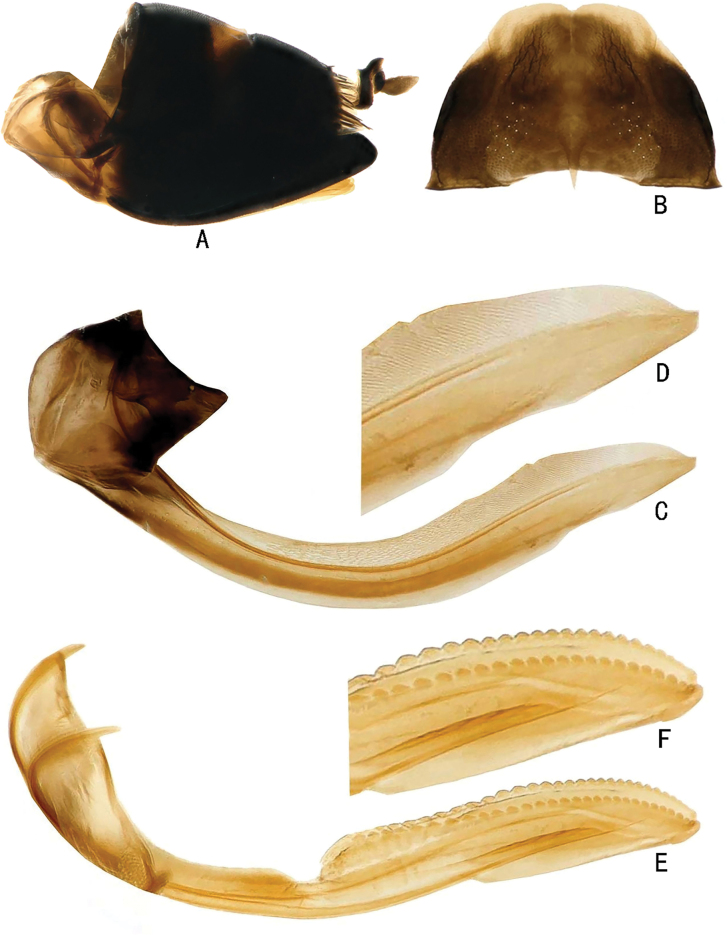
Abdominal terminalia of *Louanganastellata* (female) **A** genital capsule, lateral view **B** seventh sternite, ventral view **C** first valvula, lateral view **D** detail of sculpture of first valvula **E** second valvulae, lateral view **F** detail of sculpture of second valvulae.

**Female genitalia.** Middle width of seventh sternite about 1.6× middle length, posterior margin slightly sinuate, with a small groove in middle (Fig. 4B). Pygofer with ventroposterior margin slightly incurved (Fig. 4A). First valvulae of ovipositor with dorsal sculpture reticulate basally, with sculpturing grading to strigate and concatenate apically (Fig. 4D, C). Second valvulae with toothed distal section longer and conspicuously broader than fused basal section, more or less parallel-sided, with teeth prominent and evenly spaced, gradually becoming smaller distally, apex of teeth rounded (Fig. 4E, F).

**Figure 5.**
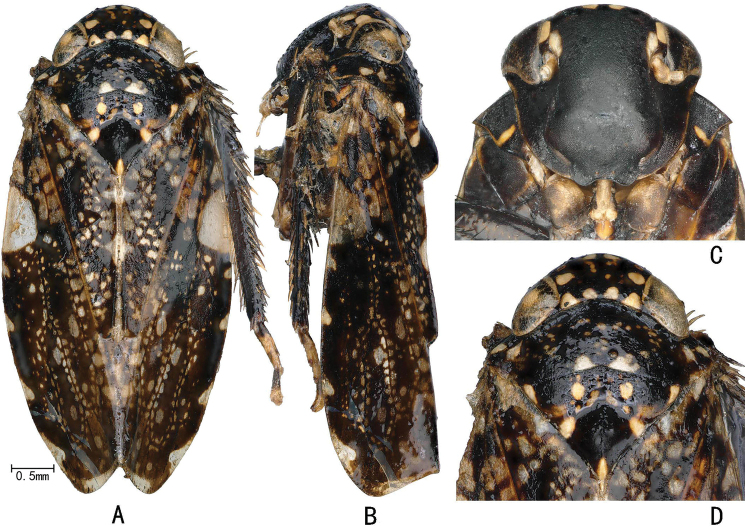
External features of *Louanganastellata* (male) **A** habitus, dorsal view **B** habitus, lateral view **C** face, ventral view **D** head and thorax, dorsal view.

**Figure 6.**
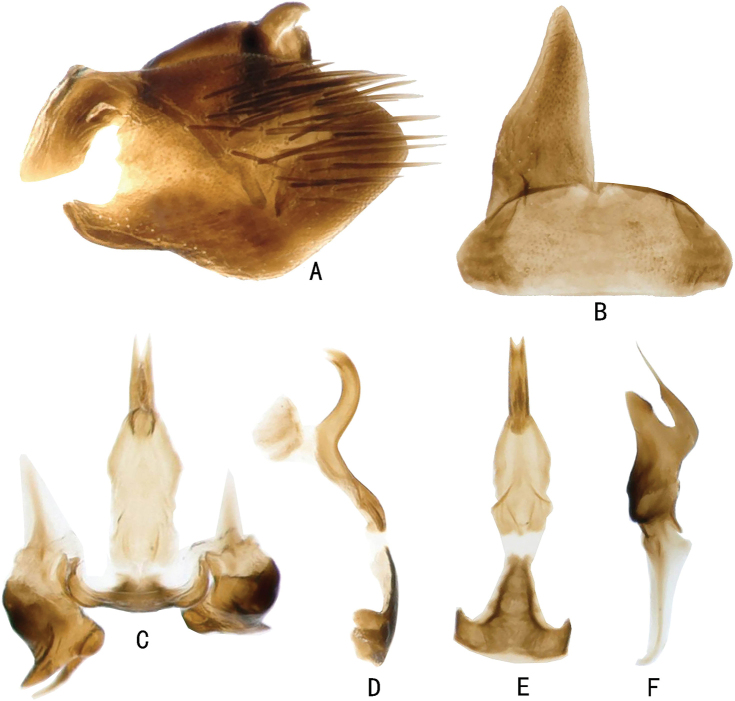
Male genitalia of *Louanganastellata***A** pygofer and anal tube, lateral view **B** valve and left subgenital plate, ventral view **C** aedeagus, connective, styles and dorsal connective, dorsal view **D** aedeagus and connective, lateral view **E** aedeagus and connective ventral view **F** style, dorsal view.

#### Measurements (mm).

Length (including tegmen): ♀, 5.67; ♂, 5.57–5.81.

#### Material examined.

**China** • **Guizhou Prov.**: 1 ♂ 1 ♀, Anlong County, Xianheping, 28 August 2012, coll. Weibin Zheng (GUGC); 1 ♂, Libo County, Maolan, 8 October 2008, coll. Qiongzhang Song (GUGC); 1 ♂, Libo County, 16 July 2015, coll. Qiongzhang Song (GUGC); 1 ♂, Wangmo County, 22 August 2012, coll. Jiankun Long (GUGC).

#### Distribution.

Laos (Louang Namtha), China (Guizhou).

## Supplementary Material

XML Treatment for Louangana

XML Treatment for Louangana stellata

## ﻿Additional information

### Author ORCIDs

Keting Duan https://orcid.org/0009-0008-8381-8890

Mick D. Webb https://orcid.org/0000-0002-1312-6142

Jichun Xing https://orcid.org/0000-0002-3738-7996

### Data availability

All of the data that support the findings of this study are available in the main text.
